# It’s About Time: The Circadian Network as Time-Keeper for Cognitive Functioning, Locomotor Activity and Mental Health

**DOI:** 10.3389/fphys.2022.873237

**Published:** 2022-04-25

**Authors:** Müge Yalçin, Annakarina Mundorf, Freya Thiel, Sandra Amatriain-Fernández, Ida Schulze Kalthoff, Jan-Carl Beucke, Henning Budde, Susan Garthus-Niegel, Jutta Peterburs, Angela Relógio

**Affiliations:** ^1^ Institute for Theoretical Biology (ITB), Charité—Universitätsmedizin Berlin, Corporate Member of Freie Universität Berlin, Humboldt-Universität zu Berlin and Berlin Institute of Health, Berlin, Germany; ^2^ Molecular Cancer Research Center (MKFZ), Medical Department of Hematology, Oncology, and Tumour Immunology, Charité—Universitätsmedizin Berlin, Corporate Member of Freie Universität Berlin, Humboldt-Universität zu Berlin and Berlin Institute of Health, Berlin, Germany; ^3^ Institute for Systems Medicine and Faculty of Human Medicine, MSH Medical School Hamburg, Hamburg, Germany; ^4^ Institute and Policlinic of Occupational and Social Medicine, Faculty of Medicine, Technische Universität Dresden, Dresden, Germany; ^5^ Institute for Systems Medicine and Faculty of Human Sciences, MSH Medical School Hamburg, Hamburg, Germany; ^6^ Department of Psychology, Humboldt-Universität zu Berlin, Berlin, Germany; ^7^ Department of Clinical Neuroscience, Karolinska Institutet, Stockholm, Sweden; ^8^ Department of Child Health and Development, Norwegian Institute of Public Health, Oslo, Norway

**Keywords:** circadian clock network, circadian medicine, circadian dysregulation, neurocognitive functioning, neurodegenerative disorders, mental health

## Abstract

A variety of organisms including mammals have evolved a 24h, self-sustained timekeeping machinery known as the circadian clock (biological clock), which enables to anticipate, respond, and adapt to environmental influences such as the daily light and dark cycles. Proper functioning of the clock plays a pivotal role in the temporal regulation of a wide range of cellular, physiological, and behavioural processes. The disruption of circadian rhythms was found to be associated with the onset and progression of several pathologies including sleep and mental disorders, cancer, and neurodegeneration. Thus, the role of the circadian clock in health and disease, and its clinical applications, have gained increasing attention, but the exact mechanisms underlying temporal regulation require further work and the integration of evidence from different research fields. In this review, we address the current knowledge regarding the functioning of molecular circuits as generators of circadian rhythms and the essential role of circadian synchrony in a healthy organism. In particular, we discuss the role of circadian regulation in the context of behaviour and cognitive functioning, delineating how the loss of this tight interplay is linked to pathological development with a focus on mental disorders and neurodegeneration. We further describe emerging new aspects on the link between the circadian clock and physical exercise-induced cognitive functioning, and its current usage as circadian activator with a positive impact in delaying the progression of certain pathologies including neurodegeneration and brain-related disorders. Finally, we discuss recent epidemiological evidence pointing to an important role of the circadian clock in mental health.

## Introduction

Life on earth has evolved around the approximately 24 h solar day. Accordingly, organisms ranging from plants to mammals, including humans, have developed an endogenous (internal) time keeping machinery–the biological (circadian) clock. The term endogenous here refers to the processes derived from an internal origin of the system itself i.e., from the organism or cell/tissue. Hence, endogenous rhythms refer to rhythms which the organism is able to generate without external input. The clock enables the timing regulation of physiology and behaviour and provides an advantage for adaptation, survival, and anticipation to foreseeable daily changes such as the light and dark cycles (Astaburuaga et al., 2019). These rhythms are circadian (*circa* = about; *dies* = a day) and show a periodicity of approximately 24 h. The timing of cellular processes, regulated by the circadian clock, is crucial for the maintenance of proper organismal homeostasis.

Increased awareness concerning the impact on health caused by perturbations in our internal time keeping machinery has strongly contributed to the growing research in the field. In 2017, discoveries on genetics and molecular mechanisms of the biological clock made over the past decades led to the award of the Nobel Prize for Physiology or Medicine to Jeffrey C. Hall, Michael Rosbash and Michael W. Young (Bargiello et al., 1984; Zehring et al., 1984; Siwicki et al., 1988; Hardin et al., 1990; Liu et al., 1992; Vosshall et al., 1994; Price et al., 1998). Although accumulating evidence points to a crucial role of the biological clock in temporal regulation and optimal functioning of physiological and behavioural processes, clinical applications of this knowledge in the maintenance of health and fighting disease remains scarce. For numerous reasons, ranging from the low number of clinical studies on chronobiology and circadian medicine to the logistic complexity of introducing circadian consideration in the planning of clinical studies and subsequent treatment routines, a limited number of clinical applications has been introduced, one of those rare examples being the use of circadian lighting solutions implemented at intensive care units (Luetz et al., 2019). It is thus timely to better understand the biological clock, as well as the consequences of its failure on human health, and develop ways for applying this knowledge in clinical practice.

**FIGURE 1:**
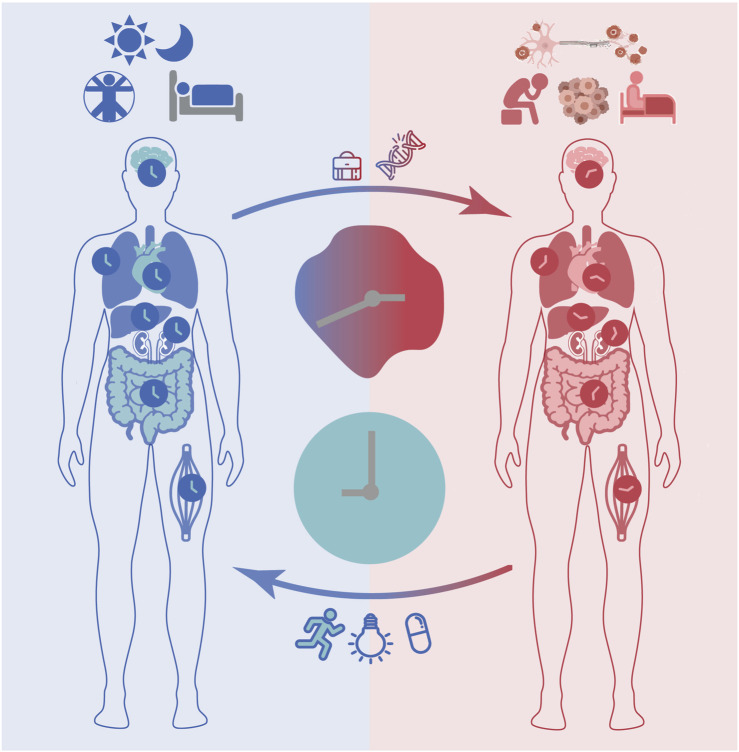
The circadian clock impacts health and disease. In response to environmental cues such as daily light/dark cycles, the master clock in the suprachiasmatic nucleus (SCN) and peripheral clocks are synchronized through complex routes of neuronal and hormonal networks. Proper functioning of the circadian clock ensures correct timing of physiology and behaviour (e.g., sleep/wake cycles) and contributes to maintaining a healthy life. Disruptions of circadian rhythms may occur due to genetic factors or life style determinants such as long-term shift work and are associated with various health complications including mood and sleep disorders, neurodegenerative diseases and cancer. Clinical interventions aiming at restoring circadian rhythms and minimizing the consequences of circadian disruption (e.g., bright light therapy, physical exercise), as well as targeting treatment optimization via timing the administration of drug compounds (i.e., chronotherapy) are currently being evaluated.

Circadian rhythms share certain characteristics, as they are: 1) self-sustained oscillations (∼24 h period) present also in the absence of external cues (defined as free running period, FRP); 2) susceptible to resetting by Zeitgebers (timing cues) as defined by pioneers in the field (Pittendrigh and Minis, 1964; Aschoff, 1965); and 3) temperature-compensated. The German term Zeitgeber (literally “time giver”) is defined as any external or environmental cue that entrains an organism’s biological rhythm to cyclic, repetitive signals to ensure the temporal regulation, adaptation and functioning of the organism. These environmental factors refer to changes in the surrounding environment such as light exposure, temperature, oxygen or nutrient availability, mealtimes, physical exercise, work schedules, and social cues as illustrated in Figure 1. Of note, although any environmental factor which can enable a 24 h rhythm may serve as Zeitgeber for other oscillators, light has been denoted as the primary and the most dominant Zeitgeber to the circadian clock (Duffy and Czeisler, 2009; Ashton et al., 2022). The underlying reason being is that light and dark cycles are responsible for all other environmental rhythms therefore serving a reliable source of information to adjust the timely regulation of process across the day (Panda et al., 2002; Roenneberg et al., 2013a). In addition, several internal signals such as daily fluctuations of hormones or metabolites in bloodstream, neurotransmitters or body temperature can also act as ‘internal timing cues’ in mammals (Dibner et al., 2010).

As mentioned above, circadian rhythms are synchronized to external light signals every day to ensure robust cyclic activity in molecular and cellular processes, which in turn regulate the timing of physiology including core body temperature, sleep/wake cycles, hormonal secretion (e.g., cortisol, melatonin and dopamine), and behaviour (e.g., physical activity and mood) (Koronowski and Sassone-Corsi, 2021) (Figure 1). Both genetic (the alterations in of clock or clock-controlled genes) and/or environmental factors (e.g., sleep disruption, artificial light exposure) may disrupt circadian rhythms (Cederroth et al., 2019; Ruben et al., 2019; Walker et al., 2020). Several studies have shown that, in the absence of environmental cues, humans have altered sleep and activity cycles (slightly longer than 24 h), and that genetic variants of certain circadian genes can affect period length (Toh et al., 2001; Xu et al., 2005; He et al., 2009) and phase of behavioural outputs (Archer et al., 2003; Yamaguchi et al., 2015). While homeostatic sleep regulation refers to the trigger to sleep, which is influenced by the duration of wakefulness, the circadian sleep regulation transmits stimulatory signals to circadian networks to promote awakening in opposition to the homeostatic drive to sleep. Thus, homeostatic sleep processes have a strong influence on the maintenance and depth of sleep. According to the well-known two-process model of sleep regulation, which suggests that, this regulation is determined based on the interaction of homeostatic processes (also known as process S), and the prior amount of sleep and fragmentation (by awakening), which is controlled by the circadian clock (process C). Sleep occurs when S approaches its upper boundary, while awakening occurs at its lower boundary (Borbély, 1982; Daan et al., 1984; Borbély et al., 2016).

Environmental cues can influence not only the period, but also the phase and amplitude (peak to trough distance) of circadian oscillations (Roenneberg and Merrow, 2007). The phase of an oscillation is defined as the timing of maximum expression and/or activity within a circadian cycle (24 h). The term “chronotype” on the other hand is used to denominate an individual’s subjective, internal timing, and provides a classification of individuals (i.e., morning larks or night owls) based on their preferred sleep-wake phases (Roenneberg et al., 2003; Roenneberg et al., 2019; Hesse et al., 2020). As a result of the different chronotypes, humans show a predisposition to be more efficient at certain activities (e.g., sports, food intake) at different times of day, and following one’s chronotype is crucial to maintain optimal functioning of the circadian system.

Continuous alteration of the timing of daily routines, for example due to shift work or travel, can result in the misalignment between internal and external time (Figure 1). The term ‘circadian misalignment’ in this context can be described as a mismatch between an individual’s circadian chronotype and their physical or social environment (e.g., light-dark cycle, school, or work times) (Roenneberg and Merrow, 2016). This means that, if circadian rhythms in all cellular clocks are synchronised to the daily cycle, as well as to each other, they are aligned. On the other hand, if throughout the body, they are termed as misaligned. The resulting disruption of circadian rhythms negatively impacts human health and has been found to be associated with various diseases including sleep disorders (Rijo-Ferreira and Takahashi, 2019), mental disorders (Walker et al., 2020), neurodegenerative diseases (Carter et al., 2021) and cancer (Sulli et al., 2019), as well as metabolic disorders such as obesity (Baron and Reid, 2015) and diabetes (Javeed and Matveyenko, 2018). Although a limited number of interventions are available a few prototypes have been used to prevent and/or reduce circadian disruption in daily activities. For example, special bio-lightening in overseas flights has been implemented to prevent misalignment of the internal clock and the geophysical time while traveling across time zones, a concept described as ‘jet lag’. Other examples include exposure to bright light in seasonal depressive disorder, a mental condition related to variation of daily light exposure across different seasons, which has been reported as an effective treatment equivalent to antidepressants (Campbell et al., 2017). Disruptions of circadian rhythms in sleep/wake cycles, blood pressure, and hormonal secretion such as melatonin have been reported in patients with mood disorders. Clinical studies have pointed to a direct association between the severity of circadian rhythm disturbances and mood disorders and further showed a positive impact on mood disorders when restoring circadian dynamics with treatments (Ruben et al., 2019; Sato et al., 2021).

As mentioned above, research in the circadian field has grown considerably over the past years, but usage of this knowledge in the maintenance of health and prevention or treatment of disease remains to be fully explored. Increased awareness of the role of circadian rhythms in health maintenance, as well as non-invasive tools to characterize the individual chronotype, also at the molecular level, are therefore needed.

In this review, we will first provide an overview of the current knowledge regarding the molecular and neuronal circuits that drive circadian rhythms in mammals, with a particular focus on humans, followed by a discussion of recent studies reporting connections between perturbations of the circadian clock and pathological development. We will highlight the link between a dysregulated clock and neurodegenerative diseases including Alzheimer’s disease (AD), Parkinson’s disease (PD), and Huntington’s disease (HD), and describe results from recent studies reporting restoration of circadian rhythms as a means to improve disease-related symptoms (Section 3.1). We will further discuss the circadian regulation of behavioural and cognitive functions, memory consolidation, and implication of loss of this regulation in mental disorders such as obsessive-compulsive disorder (OCD), major depressive disorder (MDD), and schizophrenia (Sections 3.2). We will elaborate on emerging novel aspects linking the circadian clock to physical exercise and exercise-induced neurocognitive functioning, as well as its current usage as circadian activators with a positive influence on delaying the progression of certain neurodegenerative and brain disorders (Section 3.3). Finally, in Section 3.4, we will discuss recent epidemiological studies pointing to the important role of the circadian clock in mental health with a particular focus on its link to public mental health and the transition to parenthood.

## Making Biological Time: The Molecular and Neural Circuits Mediating Generation and Functioning of Circadian Rhythms

### The Suprachiasmatic Nucleus as the Master Regulator of the Circadian System

In mammals, the circadian system is hierarchically organized. The central pacemaker of the circadian system is located in the suprachiasmatic nucleus (SCN), a small bilateral structure in the anterior part of the hypothalamus positioned above the optic chiasm. In humans, the core-molecular oscillator is reset with exposure to light in the early morning. Environmental light signals are perceived via intrinsic photosensitive ganglion cells and photic information is transmitted to the SCN via the retinohypothalamic tract. Via complex routes of neuronal and hormonal networks, the SCN entrains peripheral oscillators and modulates the period and phase of all oscillators throughout the body (Bittman, 2021).

**FIGURE 2:**
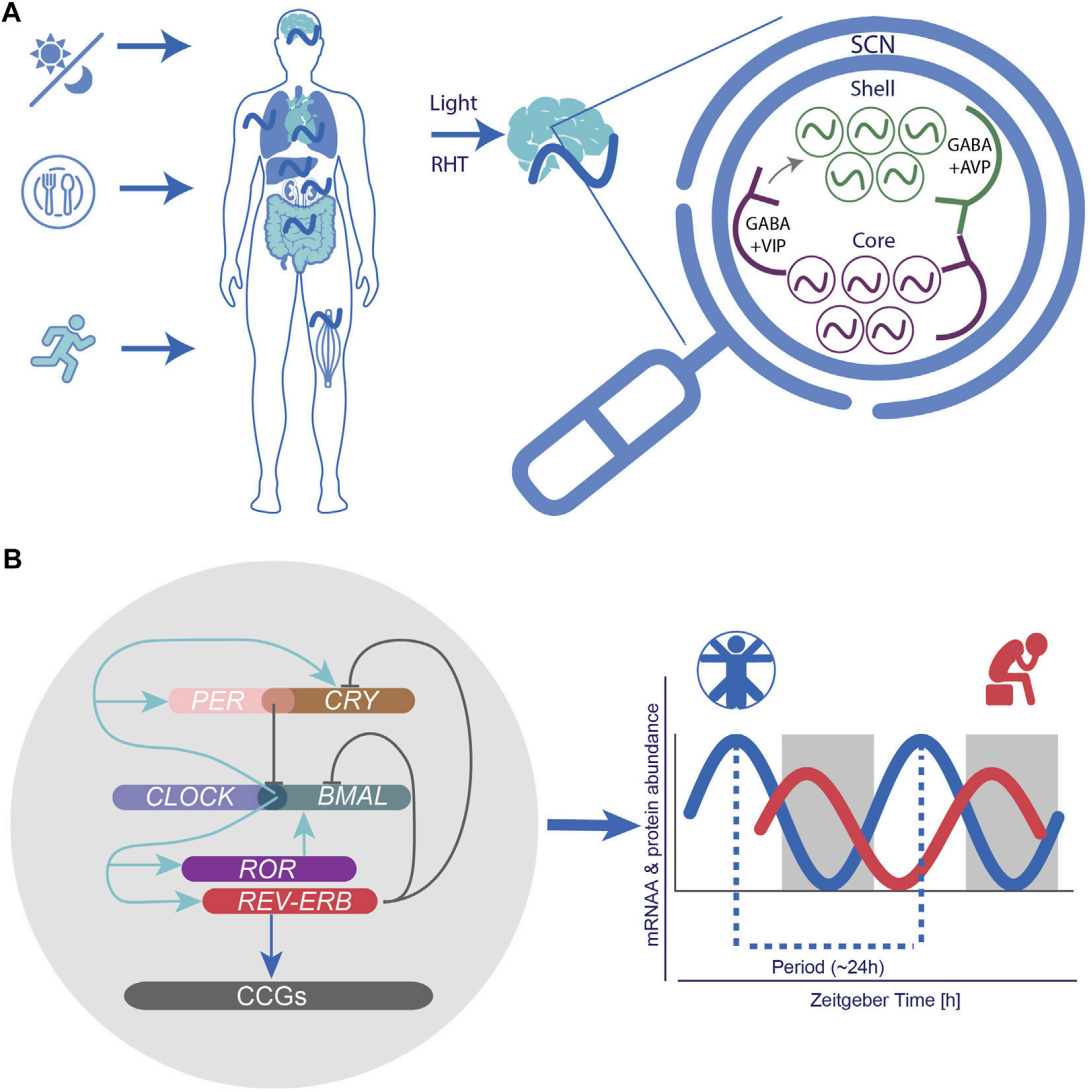
The circadian system as a neural and molecular network and implications for circadian medicine. **(A)** Following light exposure, activated intrinsic photosensitive ganglion cells transmit photic information to the suprachiasmatic nucleus SCN via the retinohypothalamic tract (RHT). The SCN synchronizes peripheral clocks throughout the body via endocrine, neuronal routes and behavioural outputs. The SCN is organized in a “core” (with VIP expressing neurons) and a “shell” region (with AVP expressing neurons), which ensure neuronal coupling. **(B)** At the molecular level the circadian network is formed by intricated self-sustained feedback loops of core-clock elements (CLOCK, BMAL, PERs, CRYs, RORs, and REV-ERBs), which drive 24-h rhythmic oscillations at the mRNA and protein level of several target genes.

The generation of time in the main pacemaker is ensured by clusters of coupled oscillators (∼20.000 neurons) spread across two SCN subregions, the core and a shell region surrounding the core, which differ in their expression of specific secreted neurochemicals (Hastings et al., 2018) (Figure 2A). The core region governs vasoactive intestinal peptide (VIP)-, calretinin, neurotensin (NT)-, and gastrin-releasing peptide (GRP)-expressing neurons (Slat et al., 2013). VIPs are the most abundant neurotransmitters and are essential for the transmission of light signals. Together with their binding partner VIP-receptor 2 (VIPR2; also known as VPAC2), found in both core and shell regions, VIP neurons regulate the rhythmic expression of other neuronal circuits and ensure the coupling of SCN neurons, as well as non-SCN brain clocks. Perturbation of VIP signalling via knockout (KO) experiments resulted in the disruption of inter-neuronal coupling and rhythmic expression of core-clock genes, and led to the dysregulation of activity and entrainment mechanisms in mouse models, pointing to an essential role of this coupling on physiological processes (Maywood et al., 2011; Edwards et al., 2016).

The shell region, on the other hand, is composed of arginine vasopressin (AVP)-expressing neurons that colocalize with Gamma-Aminobutyric Acid (GABA), a crucial neurotransmitter, which regulates excitability of neurons in the central nervous system (CNS), and plays a role in the phase resetting of other brain (non-SCN) and peripheral clocks throughout the body (Herzog et al., 2017; Mieda, 2019). AVP neurons are under direct regulation of the circadian clock and widely present in the SCN. They can substitute the function of VIP neurons, in the absence of VIP signalling (Maywood et al., 2011). The absence of AVP receptors was shown to weaken the coupling between SCN neurons, and to accelerate re-entrainment of behavioural rhythms in mice (Yamaguchi et al., 2013; Mieda et al., 2015).

In addition, the SCN plays an essential role in the entrainment of peripheral clocks (Figure 2A). It has been suggested that a bidirectional interaction may exist between the master pacemaker and other body clocks pointing to the existence of a feedback route from peripheral clocks to the SCN. Although the exact mechanisms underlying this interaction remain to be elucidated, energy homeostasis via the regulation of the hormones ghrelin (involved in short-term appetite regulation) and leptin (considered as a satiety hormone) can modify the activity of SCN neurons (Grosbellet et al., 2015). Also melatonin, a pineal gland rhythmically secreted hormone involved in the regulation of sleep and wake timing, activates its complement receptors (MT_1_ and MT_2_) in the SCN, thereby regulating the robustness of circadian rhythmic activity (Liu et al., 2016). In turn, melatonin is also under the direct control of the SCN (Cajochen et al., 2003; Zisapel, 2018; Arendt, 2019).

Interestingly, recent studies on *Bmal1* (brain and muscle ARNT-like 1) KO mice showed that besides synchronizing the SCN, light stimuli can synchronize peripheral tissues such as liver (Koronowski et al., 2019) and epidermis (Welz et al., 2019). Although peripheral clocks (e.g., liver and kidney) can maintain endogenous rhythms and be synchronized by other external cues such as feeding, ablation of the SCN in mice lead to a high variation of circadian phases among body clocks (Izumo et al., 2014). Studies on a mouse model with SCN-specific disruption of *Bmal1* have reported intact core-clock oscillations in the liver under light/dark conditions (LD: 12 h light, 12 h dark), but dampened after a few days in dark/dark (DD: 24 h dark) conditions (Husse et al., 2014). Altogether, these observations suggest that in the absence of light stimuli, other signals transmitted by peripheral clocks may contribute to the maintenance of 24 h rhythms.

### The Molecular Clock Network and Clock-Controlled Cellular Processes

At the cellular level, the mammalian SCN and other peripheral clocks are molecular oscillators consisting of self-sustained transcriptional and translational feedback loops (TTFLs). In humans, the TTFL is composed of genes and proteins, which form positive and negative feedback loops. At the beginning of each circadian cycle, circadian locomotor output cycles protein Kaput (*CLOCK*) heterodimerizes with brain and muscle ARNT-like 1 (*ARNTL*, also known as *BMAL1*) and the complex binds to the enhancer regulator sequences (E-boxes) in the promoter regions of their target genes period (*PER*s), cryptochrome (*CRY*s), nuclear receptor subfamily 1 group D member 1 (*REV-ERBα*, also known as *NR1D1*) and nuclear receptor subfamily 1 group D member 2 (*REV-ERBβ*, also known as *NR1D2*), and retinoid-related orphan receptors (*RORs)* to activate their expression. Additional interconnected feedback loops consist of positive (*ROR*s) and negative (*REV*-*ERB*s) elements and ensure the robustness and precision of the circadian oscillator (i.e., amplitude and phases) by fine-tuning the transcriptional activity of *BMAL1*. *ROR*s and *REV-ERB*s regulate the activation and repression of *BMAL1* respectively by acting on the REV response element (RRE) sequences.

In humans, at dawn, following the accumulation of *PER* and *CRY*, the PER/CRY heterodimer complex forms and suppresses BMAL1/CLOCK-mediated transcription in the nucleus (Figure 2B). Once the PER/CRY complexes are degraded, a new circadian cycle (∼24 h) is initiated (Partch et al., 2014). The molecular clock is also influenced by posttranslational modifications such as phosphorylation of PER via the casein kinase 1 (CK1) gene family in order to adjust proper timing of its activity (Etchegaray et al., 2009; Lee et al., 2011). Altogether, these interconnected elements drive and ensure the robustness of 24 h rhythmic oscillations in the expression of various additional target genes also known as clock-controlled genes (CCGs) (Figure 2B). CCGs are involved in various cellular mechanisms, including metabolism (Eckel-Mahan and Sassone-Corsi, 2013; Fuhr et al., 2018; Reinke and Asher, 2019; Sato et al., 2022), RNA processing (El-Athman et al., 2018; Yeung et al., 2018; El-Athman et al., 2019), cell cycle (Shostak et al., 2016; El-Athman et al., 2017; Farshadi et al., 2020), DNA damage response (Ando et al., 2001; Sancar et al., 2010; Preußner et al., 2017), and apoptosis (Lee and Sancar, 2011), which are often dysregulated in the context of pathological development. The circadian clock has also been reported to directly modulate the cell cycle via regulation of checkpoints such as *Myc* (Altman et al., 2015; Shostak et al., 2016), *Wee1* (Matsuo et al., 2003), *p21* (Gréchez-Cassiau et al., 2008), and *p16* (Kowalska et al., 2013; El-Athman et al., 2017).

Alterations in core-clock elements have been reported to interfere with the clock network and impact its functioning. In mice, *Bmal1* KO led to the disruption of proper circadian clock functioning and resulted in a shorter lifespan accompanied by an early aging phenotype (Kondratov et al., 2006). Rhythmic behavioural activity in mice was abolished by the simultaneous KO of *Per1* and *Per2* (Zheng et al., 2001) and *Cry1* and *Cry2* (Horst et al., 1999). Mice lacking *Rev-Erbα* continue to exhibit rhythmic behavioural output when measured via wheel running activity, but with a shorter period compared to wild type mice (Preitner et al., 2002), whereas the simultaneous loss of *Rev-erbα* and *Rev-erbβ* led to loss of rhythmicity (Bugge et al., 2012; Cho et al., 2012), evident by a drastic change in the wheel-running activity. Altogether, these results highlight the essential role of the circadian system in the regulation of cellular processes, physiology, and behaviour in mammals.

### The Disruption of Circadian Clock Elements and its Impact on Disease

Both, genetic (e.g., aberrant activity of clock or clock-controlled genes) and/or environmental factors (e.g., sleep disruption, artificial lighting) may result in the disruption of circadian rhythms and impact human health (Rijo-Ferreira and Takahashi, 2019). Moreover, the above-described circadian regulated cellular processes, including DNA repair mechanism, metabolism, and cell proliferation are frequently altered in several diseases including sleep disorders, neurodegenerative diseases (AD and PD) (Hood and Amir, 2017b; Carter et al., 2021), cardiovascular diseases (Crnko et al., 2019), obesity (Noh, 2018), diabetes (Javeed and Matveyenko, 2018), autoimmune disorders (Inokawa et al., 2020), and cancer (Hood and Amir, 2017b; Battaglin et al., 2021).

The involvement of the circadian system in the regulation of cellular pathways, which are frequently altered in neurodegenerative diseases and cancer, has been proposed using cancer cellular models (Maiese, 2017; Pacelli et al., 2019). Recently, a time course analysis using human colorectal cancer (CRC) cells with distinct clock phenotypes pointed to a link between cancer and neurodegenerative diseases involving the circadian system, and suggested the differential enrichment of genes involved in HD, AD and PD (Yalcin et al., 2020). In a follow-up study, the KO of core-clock genes in CRC cell lines was shown to disrupt rhythmic expression of cancer and neurodegeneration-related genes and led to similar alterations at the mean gene expression level, as observed in a cohort of PD patients compared to healthy controls (Yalcin et al., 2021). Circadian disturbances are among the earliest symptoms of neurodegenerative diseases such as HD, AD, and PD, and malfunctioning in the molecular mechanisms of the circadian system is thought to play a pivotal, and possibly even causal, role in their pathological development (Hood and Amir, 2017b; Carter et al., 2021). Interestingly, the usage of Zeitgebers (e.g., light) seems to support the treatment of several neurodegenerative disorders (Maiese, 2017; Carter et al., 2021).

Neurodegeneration is typically characterized by the loss of proper neuronal functioning due to excessive neuronal cell death and structural aberrations in protein configuration, such as amyloid formation as a result of accumulated alpha-synuclein in genetic forms of PD. Consequently, patients with neurodegenerative diseases show a variety of symptoms including deficits in motor control and cognition, mood disorders, or sleep dependent symptoms. A well-known example pointing to the dysregulation of the circadian system in neurodegenerative diseases is the worsening of symptoms such as increased cognitive malfunctioning, disruption of sleep profiles, failure in thermal regulation, and mental breakdowns leading to psychotic symptoms such as confusion and hallucinations in some patients during early evening hours, also known as ‘Sundown Syndrome’ (Bliwise et al., 1995; Volicer et al., 2001; Canevelli et al., 2016).

Genetic polymorphisms in clock genes have been linked to certain disease phenotypes, in particular in PD. A study by Gu and colleagues in which samples from 1394 PD patients and 1342 controls were genotyped reported that *ARNTL* (rs900147 variant) polymorphism was associated with tremor-dominant phenotype, and *PER1* (rs2253820 variant) polymorphism was linked to postural instability (Gu et al., 2015). In another large cohort study with 646 PD patients, *CLOCK* (3111T/C variant) was found to be associated with sleep disorders and deficits in motor functioning (Lou et al., 2018). In addition, the loss of circadian rhythmicity in *ARNTL* can play a role in Aβ production and plaque accumulation, an event observed in AD progression (Ma et al., 2016). In samples derived from brain tissues of post-mortem AD patients, alteration in rhythmic *ARNTL* methylation activity was reported, suggesting that perturbations in *ARNTL* functioning may contribute to behavioural and cognitive deficits in affected individuals (Cronin et al., 2017). Other studies have also pointed to the existence of a bidirectional interaction between Aβ and the clock machinery in which Aβ can cause alteration of *ARNTL* degradation, thereby disrupting clock functioning and suggesting a complex interaction between AD and the dysregulation of the circadian system (Song et al., 2015; Kress et al., 2018).

The use of interventions to revert perturbations of the circadian system was reported to ameliorate symptoms and diminish the progression of various neurodegenerative diseases. For example, exposure to bright light has been used to reverse the effect of circadian disruption in individuals with PD and AD, as well as in patients with dementia (Hood and Amir, 2017b). In a pilot study, 12 PD patients exhibiting, non-motor symptoms (insomnia and/or depression) in addition to motor PD symptoms received bright light therapy (BLT) (Willis and Turner, 2007). Patients were exposed to BLT at 1,000–1,500 Lux for 60–90 min before their usual sleep time (around 22 h for most patients) over a two-to five-week period. The results suggested an improvement of non-motor symptoms such as sleep onset and fragmentation, as well as an anti-depressant like effect improving their mood. In some patients, additional benefits were observed regarding primary PD symptoms such as slowness of movement (e.g., bradykinesia) and structural rigidity. The enhancement in psychological effects and motor symptoms was confirmed in a longitudinal follow up study, in which a larger number of patients was recruited (120 PD patients; BLT for 1 h between 20 and 22 h) (Willis et al., 2012). Motor symptoms were assessed based on three different coordination tasks and performed during daytime. Yet, an improvement was observed only in patients undergoing the light exposure protocol, and in case of withdrawal of the treatment, no improvement was observed in the test results. Another clinical study investigated the effect of BLT in a PD cohort consisting of 31 patients -receiving dopaminergic treatment (DT). Over a 2-week period, participants received 1 h BLT twice a day (morning and afternoon light exposure scheduled between 9-11h and 17–19 h) or dim-red light therapy as a control condition. Sleep quality was assessed via self-report questionnaires (Epworth Sleepiness Scale score; Pittsburgh Sleep Quality Index; the PD Sleep Scale score) (Videnovic et al., 2017). Results showed improved sleep quality metrics such as sleep fragmentation and daytime sleepiness for patients subjected to BLT. Although results from the above-mentioned studies point to a positive effect in PD symptoms resulting from circadian restoration, a characterization of the circadian clock at the gene expression level is less explored.

In a recent clinical study assessing potential alterations in circadian gene expression, hair follicles were collected from a cohort of PD patients (N = 16). The study evaluated the expression of three core-clock genes *NR1D1*, *NR1D2,* and *PER3* in comparison to a control group. Patients received BLT once a day in the evening (19–21 h) for approximately 3 months and hair samples were collected prior to and following the BLT protocol with a sampling interval of 6 h during one circadian day (Endo et al., 2020). A significant correlation between the peak expression time of *PER3* before and after BLT was documented, which revealed a significant phase delay in most patients following the BLT treatment (Endo et al., 2020). In addition, while no significant association was found between sleep related parameters using the Epworth Sleepiness Scale (ESS) (Johns, 1991), a self-report measure to assess daytime sleepiness, a correlation of sleep improvement with the delayed phase of *PER3* was reported in PD Sleep Scale 2 scores (PDSS-2), a metric to quantify the level of sleep disturbance (Endo et al., 2020).

One potential explanation concerning the role of circadian disruption in neurodegenerative diseases could relate to the change in circadian rhythms phenotype across the life span, which is mirrored in changes in individual chronotypes. Although circadian rhythms are established during early age, changes occur throughout the lifespan (Logan and Colleen, 2019). While the circadian clock of the fetus is synchronized to maternal rhythms until birth, following birth this synchrony is lost and only later re-established (within 3–5 months), once the SCN has synchronized to external light stimuli (Hood and Amir, 2017a; Logan and Colleen, 2019). Children have an early chronotype, but following puberty onset, the circadian rhythms shift. Consequently, “eveningness” tends to peak during late teens or young adulthood. With age, circadian rhythms shift back to an earlier phase, ultimately leading to the “morningness” phenotype commonly observed in healthy adults.

Aging is associated with detrimental changes in the circadian time keeping machinery (Panagiotou et al., 2021), and linked to the pathologies mentioned above. Elucidating the mechanisms underlying aging-induced circadian dysfunction is thus extremely important. In the elderly, functioning and robustness of circadian rhythms is altered, which can be partially explained by reduced sensitivity to light stimuli (Hood and Amir, 2017a), leading to a misalignment of circadian rhythms to environmental day/night cycles and subsequently to circadian disruptions in physiological and cellular processes such as poor sleep, mistiming of biochemical events such as hormonal release (e.g., melatonin), or antioxidant production which is commonly observed in the elderly, as well as in patients with neurodegenerative disorders (Musiek et al., 2013; Singh et al., 2017; Wang et al., 2018; Carter et al., 2021). Moreover, clinical studies describe 24 h rhythmic fluctuation of symptoms, particularly as PD progresses: some patients report more troublesome motor symptoms in the evening (Parkes et al., 1981; Lees, 1989); decreased response to evening doses of levodopa, a commonly prescribed anti-parkinsonian drug (Bonuccelli et al., 2000); around 70% complain of night time akinesia (Bhidayasiri and Trenkwalder, 2018); and some describe morning improvement in motor or non-motor symptoms, the so called “sleep-benefit” (van Gilst et al., 2013). It is therefore essential to characterize the individual’s internal circadian rhythm and to adjust external/internal factors in order to avoid or overcome circadian rhythm disorders. In addition, the time for certain activities (taking medication) can be optimized based on the internal timing.

**FIGURE 3:**
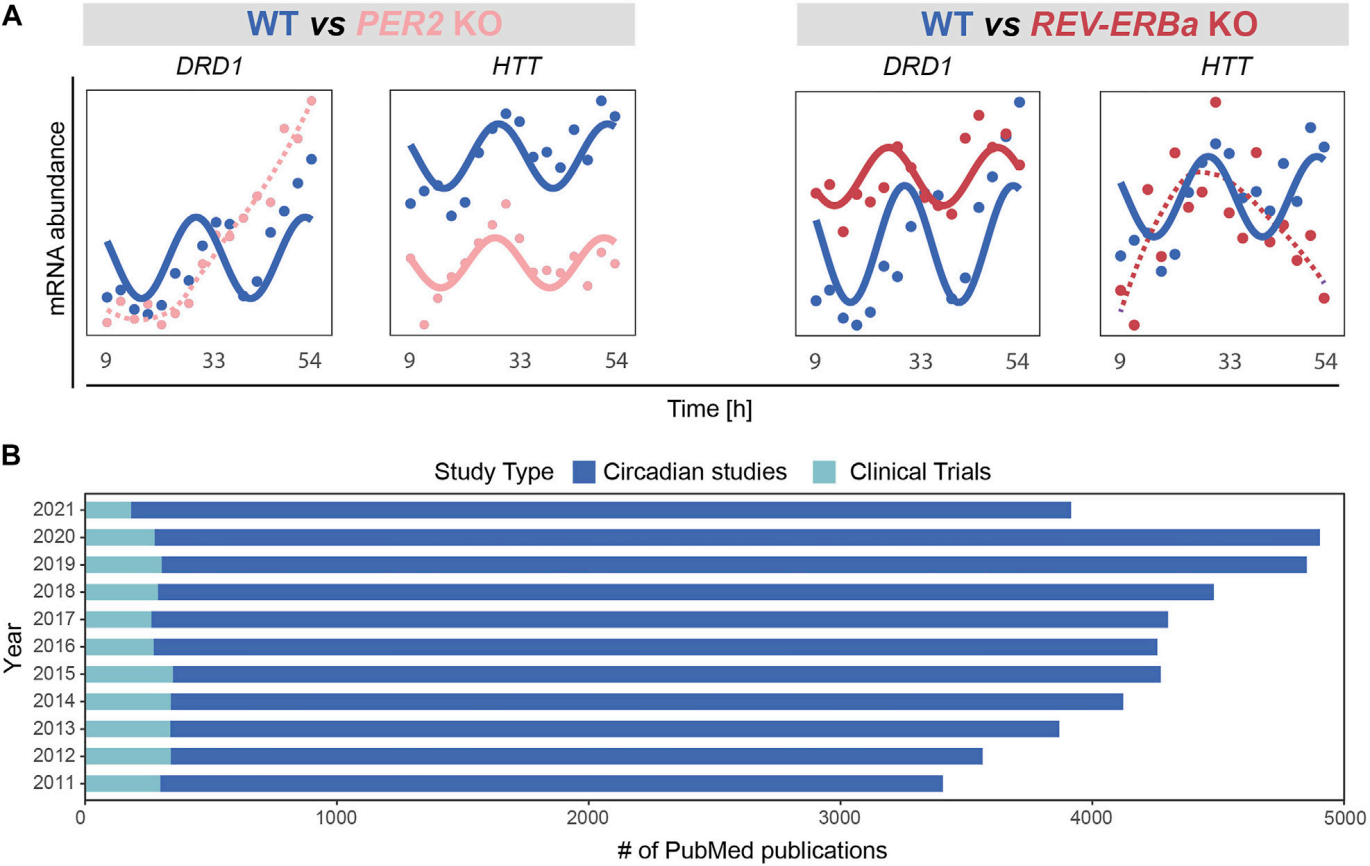
Emerging role of the circadian clock in regulation of disease associated mechanisms and its applications in basic vs. clinical research. **(A)** Perturbation of core-clock genes in an *in vitro* CRC model (HCT116 wild type (WT) and their derived *PER2* and *REV-ERB*α knockout (KO) cells, ArrayExpress: E-MTAB-9701 (Yalcin et al., 2021)) may result in complete abolishment of circadian rhythmicity and/or alteration of oscillatory properties (amplitudes or phases), as observed for *DRD1*, and *HTT*. **(B)** Number of PubMed publications in last 10 years. Studies were categorized based on research type: circadian (studies with basic research) and clinical (studies including circadian biology in a clinical study setup).

In a recent bioinformatics study pointing to molecular clock dysfunction in PD (Yalçin et al., 2021), PD patients exhibited weaker correlations in the expression of clock genes than in age and sex matched controls. In a normally functioning cell or organism, expression of clock genes is tightly ruled by intricated feedback loops and entrained by the master clock in the SCN. The reduced correlation seen in PD thus supports a dysfunction in these regulatory mechanisms. To further illustrate the potential impact of circadian disruption on neurodegeneration-related genes, we analysed the expression profile of the dopaminergic receptor DRD1 (Dopamine Receptor D1) and Huntingtin (HTT) upon perturbation of core clock genes using a recently published transcriptomics time series RNAseq data (ArrayExpress: E-MTAB-9701; (Yalçin et al., 2020)) obtained from HCT116 wild type and core-clock KO human colorectal cancer cells. *DRD1* showed a differential rhythmic expression upon perturbation of the core-clock (Figure 3A). DRD1 is tightly linked to PD, as aberrant interactions between different dopamine receptors including DRD1 in the brain were shown to be involved in l-dopa-induced dyskinesia (Lanza et al., 2021) and risk of hallucination, and circadian factors might also be involved in this interplay. Notably, HTT, whose mutation leads to HD (Li et al., 2003), also exhibited differential rhythmicity (Figure 3A). These two examples show that alterations of specific core-clock genes can indeed lead to the disruption of the normal circadian profile in relevant neurodegeneration-related genes.

The therapeutic potential of the circadian clock, also referred to as chronomedicine, gained increasing attention in recent years. Chronomedicine aims at using alternative routes for prevention, drug development, diagnostics, and treatment with a particular focus on the biological clock. A broad range of therapies and their link to circadian rhythms have been investigated mostly in animal models, including therapies against allergies, arthritis, asthma, hyperlipidemia, hypertension, cancer, and neurodegeneration. Yet, clinical studies using and aiming at validating such knowledge remain insufficient (Zhang et al., 2014). Measuring the expression of core-clock and/or clock-controlled genes involved in drug metabolism is thus extremely relevant and can be used to identify novel drug targets and to time drug administration.

To illustrate the discrepancy between basic circadian medicine-related research and published clinical studies involving circadian considerations, we carried out a PubMed search and retrieved all publications between 1 Jan 2011 and 31 Dec 2021 using the search terms: “circadian clock”; “chronobiology""; “biological clock”; “circadian rhythm”; “chronomedicine”; “chronotherapy”; “circadian medicine”; “chronotherapies”. We selected publications in English, with a full-text link available for further analysis. We first focused only on original research articles including all PubMed references published in circadian research based on human studies (including *in vitro* evidence) and excluded review papers, as well as clinical studies. Subsequently, by including only clinical studies, we identified the number of publications in which “circadian medicine” had been used as an intervention. Our analysis is in line with a previous report by Ruben and colleagues (Ruben et al., 2019) and highlights the fact that the lack of circadian considerations in study protocols remains, while it is urgently needed to allow for circadian medicine to be integrate into clinical practice (Figure 3B). For the future implementation of chronotherapy, the integration of genomics and physiological data will be necessary to understand healthy and pathological dynamics at a temporal level. In the following (Sections 3.2), we will focus on the circadian regulation of cognitive functioning in health and provide a summary of the latest evidence from mammals and how the loss of this tight regulation is implicated in mental disorders.

## Circadian Regulation of Behaviour and Cognitive Functioning

As mentioned above, biological (circadian) clocks regulate the timing of many different physiological processes including immune responses, feeding, sleep/wake cycle, reproduction, and the release of hormones such as glucocorticoids. In addition, the circadian clock is also involved in mental processes such as memory consolidation, mood, and reward (McClung, 2013; Logan and Colleen, 2019). In Section 3.1, different genetic and environmental factors influencing the circadian timekeeping system such as daylight, food intake, and social interaction are discussed. These influences might then alter certain circadian rhythms, which in turn are associated with dysfunction of cognitive processes. In this section, different behaviours and cognitive processes will be discussed in the context of circadian regulation and circadian implications associated with mental disorders.

A review of existing literature showed that in evening types, aggression and antisocial behaviour were increased compared to morning persons (Schlarb et al., 2014). Similar results were found in adults (Hood and Amir, 2018). A study involving 1,000 adult participants documented that adults identifying as evening types showed higher levels of self-reported impulsivity and anger than morning types (Hwang et al., 2016). Similarly, a study including 641 adult participants reported an association between evening type and higher levels of depressive, irritable, anxious, and cyclothymic (instable mood) behaviour, while morningness was associated with a hyperthymic (e.g., increased energy) temperament (Park et al., 2015). In a web-based study, in which participants were recruited and participated anonymously and online without any interaction with the researchers, multiple questionnaires were used to assess chronotype and temperament (Chrobak et al., 2017). A total of 618 subjects completed the Composite Scale of Morningness (CSM) (Horne and Östberg, 1976), the 72-item Sleep Wake Pattern Assessment Questionnaire (SWPAQ) (Putilov et al., 1990; Putilov and Putilov, 2006) for chronotype and the temperament evaluation of Memphis, Pisa and San Diego-Autoquestionnaire (TEMPS-A) (Akiskal et al., 2005). Interestingly, only an association between hyperthymic temperament and morningness was found, indicating that people with an earlier wake up time showed higher vigilance and wakeability (Chrobak et al., 2017).

Evidence for circadian fluctuations in aggressive behaviour has also been found in clinical populations. For instance, aggressive behaviour reported in some patients suffering from neurodegenerative disorders, such as AD and PD, shows a daily pattern: aggressive verbal or physical outburst and agitated motor behaviours were found to be increased in late afternoon and early evening (Bachman and Rabins, 2006).

However, it has to be noted that observational studies with human subjects, either in clinical or healthy samples, only provide correlational insights, whereas causal relationships between temperament or aggression and chronotype or seasonal period cannot be established. Nevertheless, the stable association reported by studies with sufficiently big sample sizes is intriguing and warrants further systematic investigation.

### Interactions of Circadian Rhythms and Food-Related Behaviours

Extensive research has been dedicated to elucidating interactions of circadian rhythms and food-related behaviour. Of note, in this line of research, food intake can be either the dependent variable, e.g., the effect of sleep deprivation on food consumption may be assessed, or an influencing factor. Studies on the latter have used different approaches to investigate the impact of food intake on sleep/wake patterns, activity levels, and more, e.g., by restricting the time periods in which food is available, or by assessing how the timing of food intake affects activity.

Scheer and colleagues investigated whether hunger exhibits a circadian rhythm in healthy participants (Scheer et al., 2013). They monitored 12 participants (6 male and 6 female) over 13 days regarding hunger and appetite ratings while controlling for sleep periods, and most of the potentially influencing environmental factors. Specifically, they controlled for meals (eucaloric, and subjects were required to consume all their food), sleep, activity, posture, room temperature, and light (by subjecting participants to a ‘forced desynchrony’ protocol). Subjects were also asked to rate their hunger, appetite, and food preferences via computerized visual analogue scales at 5 fixed times within each of the wake periods. The study revealed that hunger followed an endogenous circadian rhythm with less hunger in the morning and the greatest hunger in the evening. Similar rhythms were also revealed for appetite (Scheer et al., 2013).

Given the endogenous rhythm of hunger, the question arises whether insufficient sleep might affect hunger or eating behaviour and thereby, desynchronize this rhythm. Along these lines, Brondel and colleagues analysed whether sleep deprivation affects food consumption and physical activity (Brondel et al., 2010). A total of 12 healthy young adult men were subjected to one night of sleep deprivation and an 8 h sleep period in two different sessions and under two different conditions (sleeping at home or in the laboratory). On the following day, standardized meals were served and physical activity was measured throughout the day. After one-night of sleep deprivation, subjects consumed 22% more energy (kcal), reported greater levels of hunger before breakfast and dinner, and showed more physical activity (Brondel et al., 2010). Pleasantness of food or desire to eat were not affected by sleep deprivation (Brondel et al., 2010). Similarly, Markwald and colleagues investigated the effects of 5 days of insufficient sleep on energy intake in 16 adults (Markwald et al., 2013). Insufficient sleep increased energy needs, physical exhaustion, and food intake above the needed energy levels, and led to weight gain in women as it reduced dietary restraints (Markwald et al., 2013). Furthermore, in this study, participants ate less for breakfast but more throughout the day and after dinner. Thus, the results underline that insufficient sleep is a risk factor for weight gain and obesity. Given the increasing prevalence of insufficient sleep and obesity in modern society, researchers seek to further disentangle the impact of chronic insufficient sleep on the circadian rhythm of subjective hunger and physiological metabolites. McHill and colleagues assigned 15 young adult participants to either control (1:2 sleep:wake ratio) or chronic sleep restriction (1:3.3 sleep:wake ratio) in a 20h/day design for 6 consecutive days over 32 days (McHill et al., 2018). Subjective ratings of hunger were assessed before and after eating, and fasting hormone concentrations were measured after awakening (McHill et al., 2018). Interestingly, hunger and hormone concentration showed a circadian rhythm but did not differ between the two conditions, indicating a limited effect on the rhythmicity (McHill et al., 2018).

It must be noted that all studies mentioned above had small samples sizes and thus, the reported results should be interpreted with caution. Nevertheless, their findings underscore a circadian rhythmicity in hunger and food intake in humans, with sleep deprivation causing potential alterations in this rhythm. The influence of the sleep/wake rhythm on food intake has also been studied extensively in rats and mice (Escobar et al., 2011; DePoy et al., 2017). Studies in wildtype mice have revealed that altered food intake during normally inactive phases (in rodents during the day) shifts the body’s circadian rhythm towards meal time, leading to asynchrony between internal and external (light/dark) cycles (Escobar et al., 2011).

**FIGURE 4:**
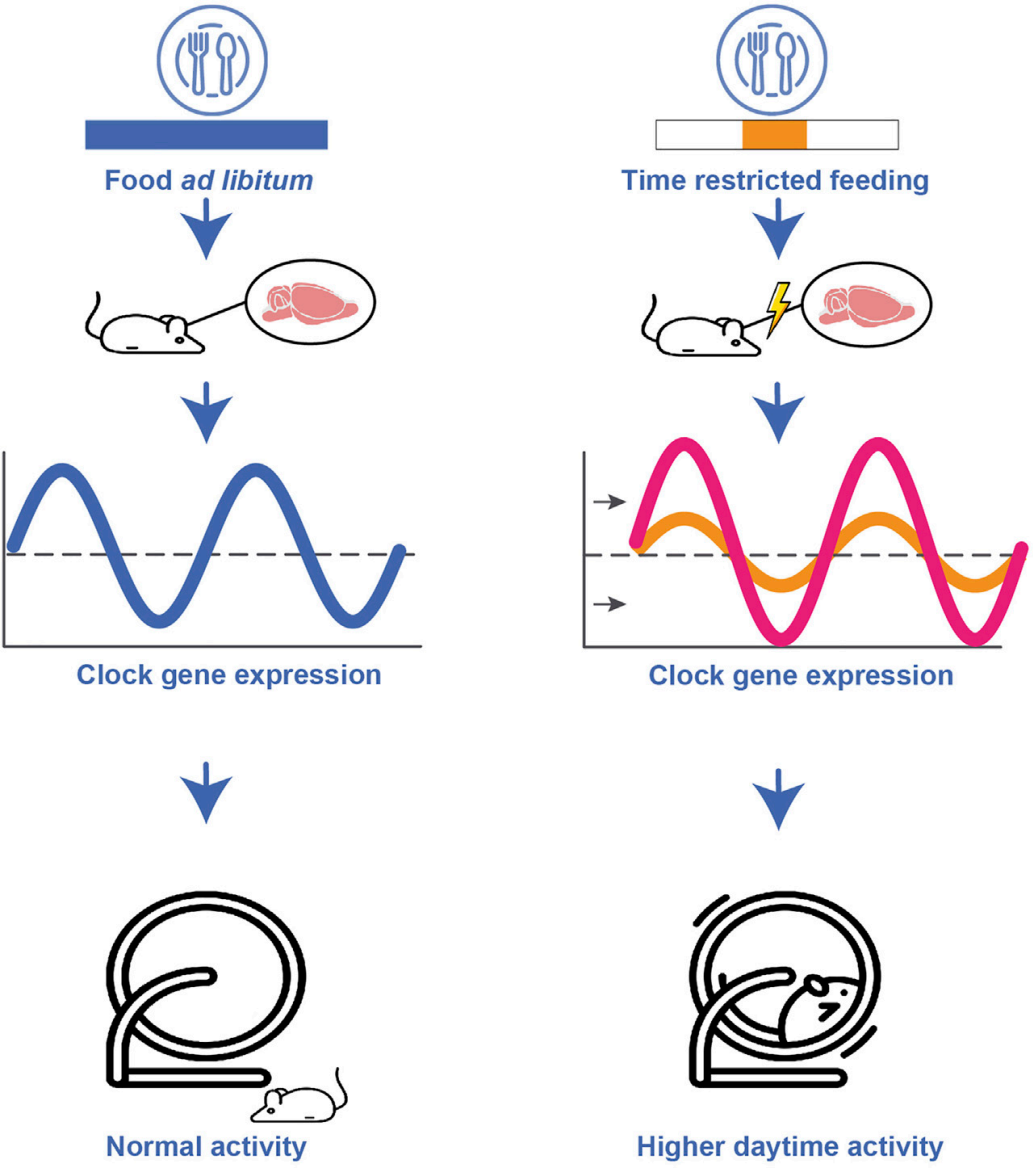
Schematic representation of the impact of altered food schedules on the circadian clock. Restricted feeding during daytime leads to a shifted, attenuated or increased clock gene expression depending on brain region. Furthermore, restricted feeding during daytime results in higher daytime activity compared to the normally low daytime activity.

Generally, some organs are more sensitive to food-induced signals than others. In rats, for example, the pineal gland reacts to the LD cycle as well as to feeding schedules (Feillet et al., 2008; Wu et al., 2008) whereas the liver is only activated by feeding schedules, and the heart and the adrenal gland, in turn, respond to signals from the SCN (Kornmann et al., 2007) (see Figure 2). Timing of food intake is thus a relevant zeitgeber, and impacts the regulating gene expression (Escobar et al., 2011). When rats are subjected to restricted feeding schedules, the daily rhythm of vasopressin release is shifted in the SCN (Kalsbeek et al., 1998) and neuropeptide Y (NPY) release in the paraventricular nucleus peaks in anticipation of food (Yoshihara et al., 1996). Moreover, studies have revealed that when rodents demonstrate food anticipatory behaviour a few hours before mealtime, hypothalamic and brain stem nuclei relevant for energy balance regulation increase their neuronal activity (measured as an increased c-Fos expression) (Angeles-Castellanos et al., 2004; Angeles-Castellanos et al., 2005). Interestingly, in the restricted feeding paradigm, food anticipatory activity in rats also leads to a peak in c-Fos expression in corticolimbic structures associated with motivation and reward (Angeles-Castellanos et al., 2007). Besides c-Fos expression, the expression of *Per1* and *Per2* was also altered by restricted feeding in these brain regions therewith detaching them from the SCN input signal reward (Angeles-Castellanos et al., 2007). Dysregulating effects of restricted feeding during daytime on the rhythmic expression of clock-genes have been found in several additional brain regions such as the hippocampus, prefrontal cortex, striatum, nucleus accumbens, olfactory bulb, and several forebrain structures involved in stress regulation, motivation, emotion, and ingestive behaviours. Notably, differential effects (i.e., increased or decreased and shifted rhythmicity in gene expression) have been found depending on brain region (see Figure 4) (Escobar et al., 2011). Studies in mice have shown that independent of caloric restriction, locomotor rhythms shift towards the time of day a highly palatable snack (meaning highly pleasant-tasting) is provided (Escobar et al., 2011; DePoy et al., 2017). For example, presenting male CBA/CaJ mice with chocolate every day at noon led to increased daytime activity, measured as running wheel activity highlighting timing of food as a factor that can alter the daily allocation of rest and activity (van der Vinne et al., 2015). This notion is particularly interesting as it might relate to our own, human, cravings for rewarding snacks that vary throughout the day and in line with our cognitive performance (Albrecht, 2011). Along these lines, a specific snack consumed at a fixed time in the afternoon might alter activity and/or performance levels. An early review article (Kanarek, 1997) shed some light on the psychological effects of snacks and meal frequency, for example concluding that nutrient intake later in the afternoon appears to facilitate subsequent performance on tasks involving sustained attention or memory. However, a number of factors have to be considered when assessing the effect of meal intake on behaviour, e.g., objective parameters such as nutrient composition or meal size, as well as individual factors such as age and gender, general activity level, or personality factors and subjective (snack) preferences (Kanarek, 1997).

Of note, the impact of subjective preferences for snack rewards on neural reward processing has recently been investigated in a series of studies with electroencephalography (EEG). These studies found that subjective preferences are complementarily represented in subjective reward valuation and in motivational value representations as reflected by distinct components of the event-related potential (ERP) (Peterburs et al., 2019b; Huvermann et al., 2021). Importantly, reward processing was not only modulated by individual factors such as subjective preferences for snacks (e.g., if one prefers salty or sweet snacks), but also by the current motivational state (e.g., the individual craving for snacks), since selective devaluation of a high preference snack by consumption to satiety decreased reward-related ERP activity (Peterburs et al., 2019b; Huvermann et al., 2021). Unfortunately, these studies did not assess circadian influences in reward processing as a function of meal intake or food preferences, nor did they acquire any measure of (cognitive) performance. However, other work has hypothesized a connection on the molecular level between circadian clock, reward system, and cognitive performance, here specifically memory (Albrecht, 2011). Circadian clock genes regulate the synthesis, function, and degradation of dopamine via specific processes (thus directly influencing monoaminergic signalling in areas of reward processing), and might thereby affect reward and reward-related behaviour (DePoy et al., 2017).

### Circadian Rhythm and Cognitive Performance

As above-mentioned, cognition is also affected by circadian rhythmicity, with effects found e.g., for attention, working memory, and executive functions, showing that performance is typically increasing during the day and decreasing during night (reviewed by (Valdez et al., 2012; Valdez, 2019)). For example, in humans, attention reaches the lowest level during night time and the early morning hours, shows better levels around noon, and the highest levels in the afternoon and evening (Valdez, 2019). Accordingly, cognitive performance can be influenced by chronotype, age, and sleep deprivation (Valdez, 2019).

On the hormonal level, melatonin might act as modulator of cognitive functioning and memory processing. Animal studies have provided evidence that melatonin decreases or inhibits long-term potentiation in the hippocampus (Wang et al., 2005; Bob and Fedor-Freybergh, 2008; Rawashdeh and Maronde, 2012). Findings is humans are less clear (Killgore et al., 2018; Rawashdeh et al., 2018). A neuroimaging study in healthy subjects did point to an effect of morning melatonin on working memory processes (Killgore et al., 2018). A decline in melatonin in the morning was associated with increased prefrontal cortex functioning. However, this effect concerned vigilance performance rather than cognitive load-dependent working memory performance. Gorfine *et al.* (Gorfine et al., 2007) investigated performance in a verbal memory task performed 2 hours after melatonin or placebo intake and failed to find differences.

Of note, clinical research in humans mainly focuses on potentially beneficial effects of melatonin treatment on cognitive and/or memory function. For instance, a recent systematic review and meta-analysis found that melatonin treatment significantly improved cognitive scores in mild levels of AD (Sumsuzzman et al., 2021). Again, effects on healthy individuals were less clear.

### Cerebellum and Circadian Rhythm

Besides brain areas underlying reward processing or memory formation and consolidation, another interesting brain structure in the context of circadian rhythms is the cerebellum. The cerebellum has traditionally been thought to primarily orchestrate motor learning and the coordination of movements as well as the timing of muscle groups to assure fluid movements (Manto et al., 2012). However, a paradigm shift has led to emphasis also on cerebellar involvement in non-motor, cognitive functions such as working memory (Desmond et al., 1997; Peterburs et al., 2019a; Peterburs et al., 2021), language and verbal fluency (Ackermann et al., 2007; Peterburs et al., 2010), error and feedback processing (Peterburs et al., 2012; Peterburs et al., 2015; Peterburs et al., 2018), and performance monitoring in general (Peterburs and Desmond, 2016).

Studies in mice have revealed that the cerebellum, like other brain regions, has a circadian oscillator relevant for the anticipation of food (Mendoza et al., 2010). The researchers analysed the effects of food restriction on the expression of clock genes in the cerebellum as well as food-anticipatory behaviours in mice with impaired cerebellar functioning (Mendoza et al., 2010). First, restricting access to food resulted in phase-shifting effects on clock gene expression in healthy mice. Second, the group investigated whether impaired cerebellar circuitry, resulting from either intracerebroventricular injection of an immunotoxin depleting Purkinje cells or from a genetic mutation (leading to impaired cerebellar circuitry and mild ataxia) changed food-anticipatory behaviours. Interestingly, mice with impaired cerebellar circuity showed reduced to no regular food-anticipatory behaviour and altered clock gene expression (Mendoza et al., 2010). These findings point towards a functional cerebellar oscillator as part of the circadian network that is crucial for mealtime anticipation (Mendoza et al., 2010). To strengthen the circadian function of the cerebellum, researchers analysed the cerebellar proteome of mice regarding rhythmic expression patterns and revealed that most proteins in the cerebellum show a rhythmical expression leading to a bimodal distribution (midday and midnight) (Plumel et al., 2019).

Another relevant aspect in the context of circadian rhythmicity is the connection between the cerebellum and the sleep-wake cycle. The cerebellum shows sleep stage-dependent activity, with disruptions changing the sleep-wake cycle (Canto et al., 2017). These changes thus can result in sleep disorders such as chronic insomnia, fatal familial insomnia, or obstructive sleep apnoea or REM sleep behaviour disorder. In line with this, sleep disorders have been associated with reduced cerebellar volume, and patients with cerebellar dysfunctions frequently suffer from sleep problems (Canto et al., 2017).

### Circadian Clock Disruption and Mental Disorders

Generally, mental disorders have been associated with a variety of alterations on the neuronal level such as volumetric reductions and neuronal structural properties (Bas-Hoogendam et al., 2020; Mundorf et al., 2021d), atypical hemispheric asymmetries (Mundorf et al., 2021c), atypical regional morphometry (Bas-Hoogendam et al., 2017), and altered neuronal gene expression in rodent animal models of mental disorders such as addiction, schizophrenia and mood disorders (Mundorf et al., 2020; Mundorf et al., 2021a; Mundorf et al., 2021b). Another aspect common to most mental disorders is a disruption of circadian rhythms, which has been investigated extensively in both animal models and clinical samples. The following paragraphs will focus on clinical research in circadian disruptions.

Individuals suffering from depression, OCD, attention deficit hyperactivity disorder (ADHD), or dementia show pronounced alterations in circadian rhythmicity in the sleep/wake cycle (also insomnia), reduced melatonin concentrations, and associated impairments in cognitive performance (Valdez et al., 2012; Valdez, 2019). Moreover, most of the prescribed medications for the respective disorders not only improve cognitive performance and affect but also influence circadian rhythms (e.g., by suppressing circadian rhythms of sleep/wake cycle, or increasing sleepiness) (Valdez et al., 2012).

In MDD and Bipolar Disorder (BD), circadian clock alterations are indicated by sleep disruptions, which represent a core/cardinal symptom and diagnostic criterion (Joel et al., 2015). In MDD, circadian clock disruptions have been studied at the molecular, cellular, physiological, and behavioural level. For example, post-mortem studies provide preliminary evidence that–in comparison to healthy control subjects–melatonin 1 receptors in the SCN and SCN GABA expression (Figure 2A) are heightened, and further that expression patterns of circadian genes in limbic and cortical brain regions are weakened in depressed patients (Li et al., 2013; Wu et al., 2013; Wu et al., 2017).

Circadian rhythms have also been investigated in the context of management of disorder management. Common treatments include total sleep deprivation, a protocol in which the patients spend 1 day (or more) without sleep to reset their sleep schedules. Results were further improved when combined with a follow-up treatment using BLT or a short phase advance protocol in several disorders (Echizenya et al., 2013), including MDD (Wirz-Justice, 2006; McClung, 2007; Kundermann et al., 2008) and Seasonal Affective Disorder (SAD) (Lam et al., 2006; Campbell et al., 2017). In meta-analyses on genetic studies, however, associations between clock genes including *CLOCK, CRY1, CRY2, PER2, NPAS2* in MDD could not be confirmed (Kishi et al., 2011; Melhuish Beaupre et al., 2020). Moreover, patients with MDD show disrupted sleep wake cycles (Germain and Kupfer, 2008), altered melatonin secretion patterns (De Berardis et al., 2015), higher nocturnal body temperatures, and decreased 24 h body temperature amplitudes when compared to healthy control subjects (Avery et al., 1982; Avery et al., 1986; Avery et al., 1999; Lorenz et al., 2019). Findings on physical activity are controversial. While some studies report preliminary evidence for lower physical activity (Difrancesco et al., 2019) and a negative correlation between depressive symptom severity and physical activity in depressed individuals (Difrancesco et al., 2021), another study found no such effects (Lorenz et al., 2019).

Like in MDD, circadian clock disruptions have been widely studied in BD. Genetic studies suggest associations between certain core clock genes such as *CLOCK, ARNTL, NPAS2, PER3* and *NR1D1* and BD–however, findings still require replication (Etain et al., 2011). Beyond that, there is preliminary evidence that melatonin profiles and *PER1* and *NR1D1* expression profiles of manic patients with BD differ from those of depressed BD patients and healthy controls (Nováková et al., 2015). Meta-analyses on circadian physiological and behavioural processes show differences between (remitted) patients with BD and healthy control subjects in total sleep time and time in bed, sleep latency, wake after sleep onset and motor activity (Geoffroy et al., 2015; Ng et al., 2015; Nováková et al., 2015; De Crescenzo et al., 2017; Meyer et al., 2020).

While most of the literature has focused on affective disorders, there is also some evidence for sleep disruptions in OCD (Coles and Stewart, 2019; Sevilla-Cermeño et al., 2019; Coles et al., 2020). To our knowledge, however, there are currently no existing studies investigating circadian clock disruptions in OCD at the molecular or cellular level. Only recently, one genetic study found an association between certain clock-controlled genes, e.g. *ARNTL* rs2278749 polymorphism, with obsessive-compulsive symptoms in a healthy control sample (Jeong et al., 2019). Moreover, findings from studies on circadian hormonal rhythms (cortisol and melatonin secretion) in OCD are still controversial regarding differences between patients and healthy control subjects (Catapano et al., 1992; Millet et al., 1999; Kluge et al., 2007). However, several meta-analyses show differences between patients with OCD and healthy control subjects in total sleep time, time awake, sleep efficiency, sleep latency, and variability in REM sleep parameters (Díaz-Román et al., 2015; Nota et al., 2015; Cox and Olatunji, 2020), strongly suggesting that circadian rhythms should be further studied in OCD.

Circadian clock disruption is also common to psychotic conditions such as schizophrenia and schizophrenia-spectrum disorders (Ashton and Jagannath, 2020). In a small sample of eleven patients with chronic schizophrenia, the expression of the clock genes *CRY1* and *PER2* has been shown to differ in comparison to healthy control subjects (Johansson et al., 2016). Beyond that, circadian gene expression appeared to be less rhythmic in patients with schizophrenia compared to healthy control subjects (Seney et al., 2019). Further, a recent meta-analysis, reported that non-acute patients with schizophrenia-spectrum disorders differ from healthy control subjects in total sleep time, time in bed, sleep latency, wake after sleep onset, and motor activity (Meyer et al., 2020).

Circadian abnormalities in schizophrenia have been hypothesized to be related to dysregulations in multiple neurotransmitter systems including hyperactivity of the dopaminergic and dysfunction of the GABAergic system (for a comprehensive review, see Monti et al., 2013). Dopaminergic dysregulation has also been linked to acceleration of the internal clock even outside of circadian effects (Davis et al., 1991), as evidenced by alterations of interval timing and duration estimation (e.g., Meck, 1996). In a study by Peterburs et al. (2013), administration of the Positive and Negative Syndrome Scale (PANSS) (Kay et al., 1987) allowed assessment of individual levels of positive and negative symptoms. When asked to estimate the time needed for a moving visual stimulus to reach a specific target position, patients underestimated movement time. This was especially prominent in patients with high PANSS positive symptoms (Peterburs et al., 2013).

It must be noted that time processing anomalies in schizophrenia such as altered interval timing or duration estimation have been investigated mostly independently from circadian rhythms. However, some researchers have argued that it is reasonable to assume that these processes may share some of their underlying mechanisms. For instance, clock genes may affect interval timing via neural oscillator networks which are mediated by dopaminergic signalling or alterations of neuronal architecture or excitability (Nicholas, 2015).

Taken together, evidence of disturbances of the circadian clock appear to occur in many mental disorders, yet the precise understanding of these alterations is currently missing, which precludes informing treatment strategies. Notably, some conditions (e.g., OCD) have been studied less extensively than others (e.g., MDD, BD). Possibly, this is due to the fact that circadian disturbances are closely linked to core symptoms only in the latter (i.e., sleep disturbances). Yet consistent evidence of sleep disturbances in conditions such as OCD indicates that a clear characterization of circadian clock alterations could also prove to be beneficial for treatment strategies in disorders in which the link between circadian clock disruption and clinical symptoms is less obvious. A goal for future research is thus to close gaps in knowledge on circadian clock disruptions in mental disorders on the molecular, cellular, physiological and behavioural level and to unravel the associated aetiology, in order to identify new starting points for prevention and early intervention.

## Circadian Rhythms, Physical Exercise and Cognitive Processes

The human brain remains in constant change, altering its functional and structural properties to adapt to changing demands (Budde et al., 2016b). This intrinsic characteristic of our brain is called neuroplasticity and allows the nervous system to adapt to environmental pressures, physiologic changes, and experiences by escaping the restrictions of its own genome (Pascual-Leone et al., 2005). Findings have shown a dynamic remodelling of grey matter throughout life characterized by the continuous creation and growth of neurons, dendrites, and new synapses as well as their elimination (Killgore et al., 2013). This plastic capacity of the brain represents the normal constant state of the nervous system through the life cycle, and is understood as a human mechanism for learning (e.g., the asset of new skills (Hötting and Röder, 2013)) growth, and general development but it could also be a cause of pathology (Pascual-Leone et al., 2005).

The natural process of aging is known to lead to functional changes in our brain that usually translates to functional and social impairments and a general cognitive decline (Bettio et al., 2017). At a cellular level, our brain plasticity is reduced as we age. There is a decrease in the creation of new synapses (Heine et al., 2004), and on average, synapses make fewer contacts, with less strength than in previous stages of life (van Praag et al., 2005). Neurobiological alterations like an increase of oxidative stress and neuroinflammation, as well as distorted intracellular signalling and gene expression, have also been associated with age-related cognitive decline (Bettio et al., 2017). However, the number of synapses, as well as the changes in their morphology, seem to oscillate not only on a long-term basis, but also on a daily cycle basis. These approximately 24 h cyclic changes in the nervous system are attributed to the circadian clock and referred to as circadian plasticity (Krzeptowski et al., 2018). Dysregulation of this system seems to affect major brain functions and may cause nervous system disorders (Logan and Colleen, 2019).

It is known that developmental changes are highly influenced by genetic factors. Over the last decades, interest in the study of the circadian typology has grown notably. Findings have pointed to individual differences on circadian rhythmic expression (of genes, hormones or biological processes such sleep or body temperature variations) affecting biological and psychological functioning in health and disease (Adan et al., 2012). Time-of-day fluctuations seem to play a role not only in cognitive functioning (Schmidt et al., 2007), but also in more specific areas such as physical performance (Drust et al., 2005).

As described in detail in Sections 3.2, in addition to governing sleep-wake cycles, circadian rhythms govern rhythms in cognitive processes like subjective alertness, mathematical ability, memory processes, and attention (Benca et al., 2009; Valdez, 2019). Executive functions (EF) are high-level cognitive processes with an essential role in the regulation of information traffic (Diamond, 2013). These functions enable us to generate the necessary sequences of actions needed to elicit the correct response to specific internal and external stimuli demands (Amatriain-Fernández et al., 2021) allowing, for example, to mentally play with ideas, to be adaptable to changing circumstances, to think before acting, to resist temptations, to stay focused or to be able to face new and unforeseen challenges (Diamond, 2013). There is general agreement that there are three core EF: inhibition, working memory, and cognitive flexibility (Diamond, 2013). It is known that EF suffer a decrease in their capacity to function due to developmental changes and ageing (Ferguson et al., 2021). However, external circumstances like stress, lack of sleep, loneliness, or lack of exercise seem to also affect these functions (Diamond, 2013).

Findings have shown that people with decreased circadian activity rhythm amplitude (peak activity) and robustness, as well as people with delayed rhythms have higher probabilities of developing dementia and mild cognitive impairment (Tranah et al., 2011). Along these lines, proper maintenance of the 24 h clock seems highly relevant for regulating processes such as neural activity, or hormonal (like the cortisol awakening response) signalling that may influence the development of disorders of the central nervous system (Logan and Colleen, 2019), as well as metabolic health (van Moorsel et al., 2016). Besides, disruptions of sleep and circadian rhythms are among the most debilitating symptoms in patients that already have neurodegenerative diseases (Fifel and Videnovic, 2021).

Aside from genetic influences, non-genetic factors such as physical exercise seem to also benefit the organization and development of some cerebral systems (Killgore et al., 2013), and even to adjust the circadian rhythm to external time cues (Yamanaka et al., 2006).

The terms physical exercise and physical activity are often used interchangeably. Nevertheless, while physical activity is defined as any bodily movement produced by the contraction of skeletal muscles that results in a substantial increase in caloric requirements over resting energy expenditure (American College of Sports Medicine, 2013), physical exercise is defined as a planned, structured, repetitive, and purposeful training exercise intervention that leads to a change in fitness (Budde et al., 2016a). Therefore, physical exercise is always a physical activity, but physical activity is not necessarily always physical exercise (Wegner et al., 2020). Exercise interventions are usually differentiated based on parameters like length, intensity, or type of exercise. While chronic interventions contain several bouts of exercise over a long period of time, acute interventions include a single bout of exercise, performed only once (Amatriain-Fernández et al., 2021). Consequently, chronic interventions can be used to evaluate the long-term effects of an exercise intervention and acute interventions are implemented to assess the immediate effects of an exercise intervention (Amatriain-Fernández et al., 2021).

### Physical Exercise and Cognitive Processes

Evidence from both animal and human studies indicates that physical exercise facilitates neuroplasticity of certain brain structures (Budde et al., 2016b). Studies with animals models (mice and rats) identified several neural mechanisms that might mediate the beneficial cognitive effects of physical exercise: the enhancement of neurogenesis, synaptogenesis, angiogenesis, and the release of neurotrophins (Hötting and Röder, 2013), like the brain-derived neurotrophic factor (BDNF), with an crucial role for synaptic plasticity, learning and memory (Vaynman et al., 2004). Besides, in the dentate gyrus of the hippocampal formation, increased blood flow and oxygenation seem to correlate with exercise-induced neurogenesis (Pereira et al., 2007). Following voluntary wheel running, increased neurogenesis in the dentate gyrus has been detected in adult mice (van Praag et al., 1999; Brown et al., 2003; van Praag et al., 2005; Kronenberg et al., 2006). Neurons generated under the running condition, were found to be functionally integrated into the hippocampal circuitry and indistinguishable from mature cells in their electrophysiological properties (van Praag et al., 2002). Following different exercise interventions that included running, adult mice showed an enhanced memory consolidation (Kohman et al., 2012) and adult rats showed a better memory abilities like learning and recalling the location of a specific platform (Vaynman et al., 2004). In adolescent rats, regular moderate aerobic treadmill exercise resulted in improvements in spatial learning abilities and an exercise-induced increase of cell density in the hippocampus and dentate gyrus (Uysal et al., 2005).

In humans, findings appear to have a similar trajectory. It has been suggested that physical exercise may trigger several processes facilitating neuroplasticity, increasing the human capacity to respond to new demands with behavioural adaptations (e.g., the allocation of greater attentional resources, the ability to process information quicker or an enhanced memory performance) (Hötting and Röder, 2013). Even though the underlying mechanisms associated with exercise-induced in the human brain remain to be elucidated (Killgore et al., 2013), some advances have been made in recent years. Exercise is thought to activate the necessary transcriptional machinery to modulate the expression of genes related to the regulation of synaptic plasticity, learning and memory using epigenetic mechanisms (Fernandes et al., 2017). This is not only relevant for neural cell differentiation, but also for experience-induced changes in human brain function and behaviour (high-order cognitive functions) (Feng et al., 2007). Physical exercise seems to modulate several factors relevant for the connection between skeletal muscle and the brain, such as neurotrophins (e.g., BDNF and plasma Cathepsin B (CTSB) levels) and oxidative stress parameters (e.g., lipid oxidation markers) that help to mitigate cognitive decline (De la Rosa et al., 2019). In this regard, recent research situates physical exercise as a mediator of neurogenesis in the hippocampus via BDNF (Liu and Nusslock, 2018). Exercise interventions have also shown to produce benefits in the physical functioning, health-related quality of life, strength, balance, and gait speed of PD patients (Goodwin et al., 2008). Recent findings have suggested that exercise improves sleep quality in people with Parkinson (Amara et al., 2020), situating it as an effective nonpharmacological intervention to improve this disabling nonmotor symptom that normally comes with the disorder.

Results from longitudinal studies have also shown that long-term physical exercise interventions (35 ± 15 years) promoted memory maintenance in middle-aged men and showed a connection between delay of age-related neurodegeneration and the neurotrophic and redox peripheral modulation produced by the physical exercise intervention (De la Rosa et al., 2019). Lower cerebral blood flow was associated with poorer memory and processing speed abilities in older adults with type 2 diabetes (Bangen et al., 2018) as well as with poorer attentional and memory capacities in adults with cardiovascular disease (Alosco et al., 2013).

Exercise has been situated as a preventive factor for age-related cognitive decline (Kronenberg et al., 2006), but the positive effects of exercise interventions on cognition are not limited to elderlies with preconditions. Findings showed that, in general, older adults who exercised throughout life exhibited less brain tissue loss than their sedentary peers (Colcombe and Kramer, 2003). Aerobic exercise interventions have produced increased hippocampal volume and enhanced cognitive functioning in different age-samples (Killgore et al., 2013). A study with overweight children found that increased cardiorespiratory fitness and speed-agility may have a positive impact on the development of different regions in the brain as well as on academic indicators and might neutralize the harmful effect caused by obesity and overweight on brain structures (Esteban-Cornejo et al., 2017). Additionally, a study with pre-adolescents showed that those with higher physical fitness levels presented greater grey matter volume in the hippocampus and performed better on cognitive tests than peers with lower fitness level (Chaddock et al., 2010). Similar results were obtained for healthy adults (Killgore et al., 2013). Further, effects of exercise seem to be longer lasting than previously thought which situates exercise as an epigenetic modulator of brain plasticity and cognition (Fernandes et al., 2017).

### Physical Exercise and Circadian Rhythms

Both, animal (Yamanaka et al., 2008; Wolff and Esser, 2012) and human studies (Yamanaka et al., 2006; Okamoto et al., 2013; Basti et al., 2021) have also found that exercise can alter circadian rhythms in behaviour and gene expression. In addition, the circadian clock seems to also have an influence on the benefits of exercise interventions pertaining to cognitive and physical performance (Atkinson and Reilly, 1996; Drust et al., 2005; Waterhouse et al., 2005; Facer-Childs and Brandstaetter, 2015b; a; Facer-Childs et al., 2018).

Studies on this topic have reported that circadian fluctuations in molecular (gene expression) and physiological (biomechanical muscle properties) parameters correlated with exercise performance (Basti et al., 2021). In a study comparing rugby players with sedentary subjects, it was found that genes related to circadian rhythms (*BMAL1*, *ROR*-α, *CRY1*, *PER2* (*p* < 0.001), *PER1* (*p* < 0.01, and *NR1D1 (p* < 0.05) were higher in rugby players, indicating that long-term exercise can increase the expression of genes related to the circadian clock. Further, exercise performance has shown peaks in the late afternoon (15–18 h) for healthy men and women (Basti et al., 2021). These authors reported that the daily fluctuations exhibited different patterns depending on the type of exercise (endurance vs. strength) and were also accompanied by fluctuation on the expression of core-clock genes and muscle tone (Basti et al., 2021).

Differences in performance have also been investigated in relation to the individual chronotype. A study by Facer-Childs and colleagues reported that cognitive and physical performance showed a significant diurnal variation when comparing early and late chronotypes. In this study 56 healthy individuals were categorized as early or late chronotypes based on the Munich Chronotype Questionnaire (MCTQ), and their performance in tasks related to psychomotor vigilance, executive functions, and isometric grip strength showed significant variations along the day. Late chronotypes performed significantly poorer, during the morning, than early chronotypes (Facer-Childs et al., 2018). In line with these results, evening-type swimmers swam 6% slower in the morning than in the evening trial, while morning-type swimmers required 5–7 times more effort to achieve the same performance results in an evening trial as obtained in a morning trial (Anderson et al., 2018). These authors established the chronotype based on the MCTQ. Their diurnal preference was assessed by the self-reported Hornestberg Morningness-Eveningness Questionnaire (MEQ) and strands of hair were collected to characterize the genotype of each participant for PER3 SNP rs228697 and PER3 VNTR rs57875989.

In sum, despite the need for more research to deep into the connections between physical exercise and circadian rhythms, the literature points to a bidirectional relation between circadian rhythms and exercise. Exercise may help to ameliorate circadian dysregulations and circadian rhythms seem to affect physical performance.

## Epidemiological Evidence Towards Circadian Regulation of Mental Health

Epidemiological studies have established the role of the circadian clock and circadian misalignment as a factor in physical and mental health. The term circadian misalignment traditionally refers to a mismatch between an individual’s circadian chronotype and the physical or social environment (e.g., light-dark cycle, school, or work times) (Roenneberg and Merrow, 2016). In the general population, “social jet lag” represents a proposed cause of circadian misalignment. Social jet lag refers to the discrepancy between the duration of sleep during work days and non-work days (e.g., weekends) (Wittmann et al., 2006). The social Zeitgeber Theory associated with mental disorders posits that stressful life events may give rise to alterations of circadian dynamics at the cellular and physiological levels (e.g., disrupted sleep-wake cycles). As a result, affected individuals are more prone to mood-related incidents (Ehlers et al., 1988; McClung, 2013). Epidemiological studies further highlighted the common nature of social jet lag, indicating that most individuals demonstrate variations in wake-sleep times between workdays and free days, with up to 87% of the day-working population suffering from social jet lag (Roenneberg et al., 2003; Roenneberg et al., 2007; Roenneberg et al., 2012). For instance, discrepancies between the circadian chronotype and the socially determined opportunity for sleep can arise when ‘normal’ chronotypes are employed in shift work schedules or when extreme chronotypes have to comply with conventional work hours. Extreme chronotypes (i.e., preference for either early or late sleep/activity) may evolve from a combination of genetic predisposition, age, and Zeitgeber conditions (Carskadon et al., 2004; Roenneberg et al., 2004; Roenneberg et al., 2013b; Hsu et al., 2015). The need to conform to conventional work times makes evening chronotypes (i.e., preference for later sleep/activity) more susceptible to social jet lag (Duffy et al., 2001; Mongrain et al., 2006).

Besides the immediate repercussions, which pertain to sleep disturbance and/or daytime sleepiness, social jet lag has been linked to metabolic functioning, smoking, alcohol and caffeine consumption (Wittmann et al., 2010; Roenneberg et al., 2012), as well as an increased risk for depression (Drennan et al., 1991; Chelminski et al., 1999; Gaspar-Barba et al., 2009; Kim et al., 2010; Kitamura et al., 2010; Levandovski et al., 2011; Merikanto et al., 2013). Although epidemiological research has demonstrated that in the general population, chronotype follows a normal distribution varying by gender and age, societal changes such as the increase in shift work contribute to circadian misalignment and sleep disruptions. To this end, the majority of studies has incorporated methodologies focused on shift work and jet lag, utilizing questionnaire (i.e., self-report) data rather than genetic or molecular profiling to determine chronotypes.

Over the last decades, societal changes have sparked an increase in shift work with currently at least 20% of the population’s employment based on shift work schedules (Baron and Reid, 2014). Shift work typically refers to work schedules that greatly overlap with primary sleep times, i.e., where at least 50% of work falls between 10 p.m. and 6 a.m. (Baron and Reid, 2014). Compared to non-shift workers, night and early morning workers (i.e., start before 6 a.m.) experience significant sleep time reductions of between 1 and 4 h per day (Knauth et al., 1980; Åkerstedt, 1998). As a consequence, shift work is linked to reduced sleep duration and sleep quality (Drake et al., 2004). Further, associations between shift work and various negative physical and mental health outcomes, such as cancer, cardiovascular disease, obesity, diabetes, reproductive health complications, and memory difficulties have been established (Nurminen, 1998; Labyak et al., 2002; Knutsson, 2003; Drake et al., 2004; Gumenyuk et al., 2010; Esquirol et al., 2011; Gumenyuk et al., 2014; Ohlander et al., 2015; Reutrakul and Knutson, 2015; Wong et al., 2015). These adverse health outcomes may be attributed to a complex combination of maladaptive health behaviours (e.g., smoking, alcohol consumption, healthy food restrictions, feeding patterns), chronic sleep deprivation, circadian misalignment, and increased nocturnal light exposure (Baron and Reid, 2014; Abbott et al., 2015).

A hypothesized mechanism behind the link between shift work and cancer in particular has been exposure to light at night (LAN). According to the LAN hypothesis, reduced levels of the endogenous hormone melatonin may be at the centre of this link. Because melatonin is primarily produced at night and sensitive to light suppression, the LAN hypothesis proposes that the transformation of normal cells into cancer cells (i.e., carcinogenesis, oncogenesis) is reinforced by exposure to light at night. Although a clear dissection of cause and effect is difficult given the complex impact of modern lifestyles and the fact that circadian misalignment is associated with many pathologies unrelated to melatonin, epidemiological evidence for the link between LAN and cancer is strong (Hansen, 2001; Schernhammer et al., 2001; Schernhammer et al., 2006; Flynn-Evans et al., 2009; Grundy et al., 2013; Hurley et al., 2014). In fact, evidence in this area provoked the World Health Organization (WHO) to include shift work as a potential carcinogen in 2007 (WHO, 2011).

Given the aforementioned impact of the circadian rhythm and misalignment thereof on mood and interpersonal behaviours, it is almost surprising that only a limited amount of research has focused on the role of circadian rhythm during the transition to parenthood. It is by now well recognized that the perinatal period (i.e., from pregnancy to around 12 months following birth) marks a time of high vulnerability for mental health complications for (expectant) parents, with adverse outcomes for mothers, fathers, and their offspring (Thiel et al., 2021b; Knappe et al., 2021). In the first weeks following birth, for instance, up to 85% of women will experience deteriorations in mood. This so-called “postpartum blues” has been identified as a predictor of postpartum depression (Beck, 1996), which affects around 13–19% of women (O'Hara and Swain, 1996; Gavin et al., 2005) and has been documented as the most frequent complication of childbirth (Moses-Kolko and Roth, 2004; Moore Simas et al., 2019). The majority of research on parental perinatal mental health has focused on symptoms of depression (O'Hara and Swain, 1996; Eberhard-Gran et al., 2003; Paulson and Bazemore, 2010; Junge et al., 2017; Garthus-Niegel et al., 2020; Thiel et al., 2020b), anxiety (Eberhard-Gran et al., 2003; Polte et al., 2019; Thiel et al., 2020a), and post-traumatic stress disorder (Garthus-Niegel et al., 2013; Garthus-Niegel et al., 2017; Garthus-Niegel et al., 2018; Thiel et al., 2018; Dekel et al., 2019; Thiel and Dekel, 2020; Thiel et al., 2021a; Kress et al., 2021). Whereas initially, maternal mental health was at the core of this research area, increasing attention has been paid to fathers’/partners’ perinatal mental health as well (Kress et al., 2019; Thiel et al., 2020b; Garthus-Niegel et al., 2020; Kress et al., 2021; Asselmann et al., 2022a; Asselmann et al., 2022b).

During pregnancy, alterations in women’s sleep patterns and quality with adverse impacts on mood have been documented to begin as early as during the first trimester (Ross et al., 2005; Bei et al., 2015). Over the course of pregnancy, sleep duration and efficiency decrease and sleep quality is further reduced (Hedman et al., 2002; Adler et al., 2021). Further, over 40% of women experience insomnia during the first trimester of pregnancy, increasing to over 60% by the third trimester (Román-Gálvez et al., 2018). Following birth, self-reported deteriorations in sleep quality, efficiency, and duration continue for both, mothers and fathers (Lee et al., 2000; Kang et al., 2002; Montgomery-Downs et al., 2013; Obeysekare et al., 2020). Compared to the time before pregnancy, daily sleep duration has been found to be reduced by 62 min for women and 13 min for men during the first 3 months following birth (Richter et al., 2019). Importantly, deteriorations in sleep quality and duration with onset during the transition to parenthood may not fully recover for years following birth (Lee et al., 2000; Richter et al., 2019).

The potentially crucial role of sleep disruptions during the transition to parenthood in mental health may be of interest for instance with regards to postpartum psychosis, which affects 0.1–0.2% of women following childbirth. Because around 20% of women with a history of bipolar disorder experience manic episodes, which often characterize postpartum psychosis, and because those experiencing postpartum psychosis frequently continue to report symptoms of bipolar disorder later on, it has been proposed that postpartum psychosis may be a manifestation of bipolar disorder triggered by childbirth (Ross et al., 2005; Lewis et al., 2016). Postpartum psychosis may be triggered by factors also related to mania, with disruptions of sleep and the circadian rhythm as the most common triggers being (Jackson et al., 2003; Lewis et al., 2016). Because symptoms of postpartum psychosis typically begin in the immediate perinatal period (Heron et al., 2008), the period with the greatest sleep disruption (Beebe and Lee, 2007), sleep and circadian rhythm disruptions may represent risk factors for the development of postpartum psychosis in those at risk (Lewis et al., 2016). Although the link between disruptions in sleep and circadian rhythm and postpartum psychosis is often hypothesized, empirical evidence is still limited. Nonetheless, a study frequently cited in this context by Sharma and colleagues documented that postpartum psychosis was linked to night deliveries and longer labor duration, pointing towards the crucial role of sleep disruption in the development of postpartum psychosis (Sharma et al., 2004).

Despite extensive investigation of parental sleep patterns and quality during pregnancy and following childbirth as well as its relationship with mental health complications (Deforges et al., 2021), only little is known about parental circadian rhythm during this time. Initial evidence suggests that parenthood is linked with an early chronotype (Sládek et al., 2020). Further, parental circadian rhythm amplitude may be decreased following birth (Wulff and Siegmund, 2000), which can be observed as a result of irregular sleep-wake patterns or disturbance and may accumulate to circadian rhythm sleep disorder (Zee and Vitiello, 2009). The primary decline in amplitude ensues immediately following birth as a result of infant care during the night. Longitudinal studies of women following birth document worsening of amplitude reduction during the first postpartum weeks (Nishihara et al., 2002; Matsumoto et al., 2003). Although research pertaining to circadian rhythm chronotypes in relation to mental health vulnerability during the transition to parenthood is scarce, initial evidence suggests that evening chronotypes may be at an increased risk for psychiatric symptoms (Obeysekare et al., 2020).

Given the toll of pregnancy and the transition to parenthood on sleep-wake patterns, sleep quality, and efficiency resulting from infant and child care needs, the perinatal period offers a wide range of avenues for future research pertaining to the role of the circadian rhythm in mental health. Besides the vulnerability for mental health complications, the transition to parenthood is characterized by changes regarding employment, work schedules, as well as work division among couples. Large epidemiological studies have been implemented to address these issues (e.g., Kress et al., 2019). Longitudinal, epidemiological investigations following couples and children from pregnancy throughout the years following birth will offer the opportunity to gain new insights into the link between the circadian rhythm and long-term mental health in both women and men, as well as into early mechanisms involved in the development and manifestation of the human circadian rhythm from the time of birth.

## Conclusion and Perspectives

Timely regulation of cellular, physiological and behavioural processes plays a fundamental role in the, often subtle, border between health and disease. In this review, we focused on the current knowledge regarding proper functioning of the circadian system in healthy individuals and the disruption of circadian regulation observed in numerous neurodegenerative and mental disorders.

Given the broad influence of circadian rhythmicity and circadian clock genes on multiple cognitive domains such as attention, memory, or reward processing, as well as natural factors affecting daily rhythms such as dietary schedule and the sleep/wake cycle, it is not surprising that disruptions in our internal clock can result in mental health problems. Indeed, epidemiological studies have established circadian misalignment as a prominent risk factor in physical and mental health, and highlight the risks associated to social jet lag, which affects the vast majority of the day-working population. Aside from the immediate repercussions, pertaining to sleep disturbance and/or daytime sleepiness, social jet lag has been linked to adverse health behaviours and other mental health complications. Societal changes such as the increase in shift work appear to exacerbate circadian misalignment and sleep disruptions.

Given these increasing environmental risk factors such as shift work, circadian misalignment, and personal meal schedules (e.g., eating high caloric meals late in the evening), the impact on mental health can no longer be disregarded. This is particularly relevant given the growing body of research, linking several mental disorders to disrupted circadian rhythmicity. On the genetic level, several studies have found altered gene expression of clock related genes in patients suffering from mental disorders, further underlining the important role of the internal clock for mental health. However, is has to be noted that even though the association between disrupted circadian rhythms and some mental disorders, especially mood disorders, is well investigated (Walker et al., 2020), this association remains to be disentangled for many other disorders. The usage of external zeitgebers (e.g., light or physical exercise) are a promising approach for enhancing and/or re-setting circadian rhythms. This seems to efficiently contribute to diminish deterioration of neurocognitive functioning.

Circadian rhythms have been shown to influence both cognitive and physical performance. Performing an analysis of circadian rhythms in the health domain could facilitate the design of highly personalized exercise interventions. These interventions could act as preventive mechanisms to delay the onset of disorders related to circadian disruption. This combined effort between research on exercise and circadian rhythms could have a positive impact on the benefits of exercise in the prevention and treatment of several mental disorders. However, a crucial point in administering the “exercise polypill” is the dosing and, in turn, the prescription of the physical intervention (Herold et al., 2020). The answer to questions like what type of exercise is the most influenced by circadian rhythms or what type of exercise is the best intervention for preventing or treating a determined pathology needs to be established (Gronwald et al., 2018; Gronwald and Budde, 2019; Gronwald et al., 2019; Gronwald et al., 2020; Herold et al., 2020). In future studies, the inclusion of a sham condition might help to elucidate some of these open questions (Budde et al., 2018).

Although the impact of circadian rhythm and its misalignment on mood and behaviour is well known, it is surprising that only a limited amount of research has focused on the role of circadian rhythm alterations during the transition to parenthood. For (expectant) parents, the perinatal period marks a time of, high vulnerability to mental health complications, as well as severe sleep disruptions. Despite extensive investigation of perinatal parental sleep patterns and quality and its relationship with mental health complications, only little is known about disrupted parental circadian rhythm during this time, with initial evidence suggesting that evening chronotypes may be at an increased risk for psychiatric symptoms (Obeysekare et al., 2020). The perinatal period thus offers a wide range of avenues for future research. Longitudinal, epidemiological investigations following couples from pregnancy throughout the years following birth will offer the opportunity to gain new insights into the link between the circadian rhythm and long-term mental health, as well as into early prenatal mechanisms involved in the development and manifestation of the human circadian rhythm.

Thus, a new area in the medical field, circadian medicine, is slowly emerging and time-of-day adapted therapies personalized to the patient’s circadian rhythm might provide better therapeutic outcomes than commonly applied therapies. However, clinical applications of circadian intervention remain limited due to obstacles such as a current lack of a sufficient number of studies, logistic difficulties in implementing circadian treatment regimens into clinical routines, and variations of results pertaining to the desired effect of chronotherapy in comparison to standard treatment due to demographic factors such as age or gender. To overcome these obstacles, larger cohorts and a more precise stratification of patients are needed before circadian medicine can be used in clinical practice. In this regard, the combination of genomics and physiological data holds great potential for the future direction of circadian medicine.

Investigating not only disorder-specific, but also symptom-specific alterations in the circadian clock might render new insights into the aetiology and implications of mental (and other) disorders and could set the grounds to improve mental health by considering the circadian rhythm. Although research in the circadian field has grown considerably over the past years, as pointed out in our review a discrepancy between basic chronobiology research and the usage of this knowledge in clinical practice, for maintenance of health and prevention and treatment of disease is obvious. By now we know that time matters, and it is about time that we use this knowledge.

## Glossary

AD: Alzheimer’s disease
ATHD: Attention deficit Hyperactivity disorder
AMT: Anticipation of movement task
AVP: Arginine vasopressin
BD: Bipolar disorder
BDNF: Brain-derived neurotrophic factor
BLT: Bright light therapy
BMAL1: Brain and muscle ARNT-like 1
CNS: Central nervous system
CSM: Composite scale of morningness
CLOCK: Circadian locomotor output cycles protein Kaput
CRC: Colorectal cancer
CTSB: Plasma Cathepsin-B
DD: Dark/Dark
DRD1: Dopamine receptor D1
EEG: Electroencephalography
ERP: Event-related potential
ESS: Epworth sleepiness scale
FRP: Free running period
HD: Huntington’s disease
HTT: Huntingtin
KO: Knockout
LAN: Light at night
LD: Light/dark
MDD: Major depressive disorder
NPY: Neuropeptide Y
NR1D1: Nuclear receptor subfamily 1 group D member 1
NR1D2: Nuclear receptor subfamily 1 group D member 2
OCD: Obsessive compulsive disorder
PANSS: Positive and negative syndrome scale
PD: Parkinson’s disease
PDSS-2: Parkinson’s disease sleep scale 2 scores
**REV-ERB** ***α***: nuclear receptor subfamily 1 group D member 1
REV-ERBβ: nuclear receptor subfamily 1 group D member 2
ROR: Retinoid-related orphan receptors (RORs)
SAD: Seasonal affective disorder
SCN: Suprachiasmatic nucleus
SWPAQ: Sleep wake pattern assessment questionnaire
TEMPS-A: Temperament evaluation of Memphis, Pisa and San Diego-autoquestionnaire
TTFL: Transcriptional and translational feedback loops
VIP: Vasoactive intestinal peptide
